# An evaluation of the molecular mode of action of *trans*-resveratrol in the *Porphyromonas gingivalis* lipopolysaccharide challenged neuronal cell model

**DOI:** 10.1007/s11033-020-06024-y

**Published:** 2020-12-08

**Authors:** Bojlul Bahar, Sim K. Singhrao

**Affiliations:** 1 International Institute of Nutritional Sciences and Food Safety Studies, School of Sport and Health Sciences, University of Central Lancashire, Preston, PR1 2HE Lancashire UK; 2 Brain and Behavior Centre, Faculty of Clinical and Biomedical Sciences, School of Dentistry, University of Central Lancashire, Preston, UK

**Keywords:** Metabolic inflammation, Neuron, NF-kB, Oxidative stress, Reactive oxygen species, LPS, Biochemical pathways

## Abstract

*Porphyromonas gingivalis* triggers a range of innate immune responses in the host that may contribute to the development of periodontitis and dementing diseases including Alzheimer’s disease (AD). This study aimed to assess the mode of action of trans-resveratrol in modulating the *P. gingivalis* lipopolysaccharide (PgLPS) induced metabolic inflammation in a neuronal cell model. Confluent IMR-32 neuroblastoma cells were treated with trans-resveratrol from *Polygonum cuspidatum* in the presence or absence of PgLPS. The abundance of messenger ribo-nucleic acid (mRNA) transcripts of a panel of 92 genes was quantitatively assessed through targeted transcriptome profiling technique and the biochemical pathways affected were identified through Ingenuity Pathway Analysis. Gene expression analysis revealed that trans-resveratrol down-regulated the mRNA of multiple gene markers including growth factors, transcription factors, kinases, trans-membrane receptors, cytokines and enzymes that were otherwise activated by PgLPS treatment of IMR-32 neuroblastoma cells. Pathway analysis demonstrated that the cellular oxidative stress caused by the activation of phosphoinositide-3-kinase/Akt1 (PI3K/Akt1) pathway that leads to the production of reactive oxygen species (ROS), chronic inflammatory response induced by the activation of nuclear factor kappa-light-chain-enhancer of activated B cells (NF-kB) pathway and nutrient utilization pathways were favourably modulated by trans-resveratrol in the PgLPS challenged IMR-32 cells. This study demonstrates the potential of trans-resveratrol as a bioactive compound with multiple modes of intracellular action further supporting its therapeutic application in neuroinflammatory diseases.

## Introduction

Alzheimer’s disease (AD) is a neurodegenerative disease characterized by the presence of insoluble amyloid-beta (Aβ) deposits called plaques and intraneuronal neurofibrillary tangles together with clinical signs of a deteriorating memory. AD has an inflammatory component that often follows the development of the AD hallmark lesions. The microbial infections (bacteria/viruses) are implicated in the pathophysiology of AD and their relevance is the subject of current intensive research. We have reported the associations of *Porphyromonas gingivalis* lipopolysaccharide (PgLPS) in the brains of AD patients [1] and others have recently confirmed the presence of this bacterium in the brains of AD patients [2]. Further proof of concept studies in which, *P. gingivalis* was inoculated into the mouths of apolipoprotein E knock-out (APOE^−/−^) mice demonstrated *P. gingivalis* bacterial DNA and LPS had entered the brains of APOE^−/−^ mice [3], where the infection caused a significant increase in oxidative stress [4].

To understand the precise role of *P. gingivalis* in the development of hallmark lesions of AD, numerous studies performed oral infections with *P. gingivalis* or injected its lipopolysaccharide (LPS) in mice have supported the mechanism of inflammation that deteriorates further with aging [5, 6]. Studies investigating either *P. gingivalis* or its outer membrane protein, PgLPS in vitro and in vivo [2, 3, 5, 7–9] suggest that this bacterium and its virulence factors play an important role in the pathophysiology of both periodontitis and AD. PgLPS is a potent immunogenic molecule that can alter immune homeostasis through altering the levels of the amyloid precursor protein, the parent protein of the Aβ plaque lesion in AD. AD is a disease in which advancing age is a major risk factor and consequently, has the underlying inflammatory component deeply rooted in the innate immune system and metabolic inflammation. Hence, the precise role of the interplay of the innate immune and metabolic responses need to be better understood.

Resveratrol (3,4′,5-trihydroxy-trans-stilbene) is a bioactive polyphenol naturally produced in plants such as grapes, berries, peanuts, tea and the root of *Polygonum cuspidatum* [10]. Trans-resveratrol has been reported to have numerous health benefits including anti-inflammatory, anti-diabetic, anti-oxidant and neuroprotective, and has been traditionally used in oriental medicine [11, 12]. Growing evidence suggests that resveratrol has antimicrobial properties against a range of bacterial species, viruses and fungi [13]. Resveratrol inhibits the expression of virulence factor and interferes in biofilm formation where *P. gingivalis* is a keystone pathogen [14]. Resveratrol is a lipophilic polyphenol that can easily pass through the blood–brain barrier and hence is of particular interest for its bioactivity in neuronal health [15]. The neuroprotective role of trans-resveratrol against cellular oxidative damage has been previously reported [16]. In addition, several resveratrol derivatives have also been tested for their potential benefits in preventing neuronal cell death due to Aβ deposition (an antimicrobial peptide in AD brains) taking place in the brain [17].

*P. gingivalis* can contribute to systemic and intracerebral pools of Aβ as well [8, 9]. It is therefore plausible to suggest that *P. gingivalis* oral infection due to severe periodontitis has metabolic implications to an individual’s peripheral and brain health. Neurons in the brain are vulnerable to oxidative stressors and a report by Kim et al. [18] suggested that resveratrol has a neuroprotective role by directly blocking the cellular oxidative stress caused by the release of reactive oxygen species (ROS). Resveratrol, despite being a highly bioactive molecule with a range of protective effects in the chronic inflammatory diseases, its therapeutic use in neuroinflammatory diseases particularly in the context of PgLPS induced oxidative stress and innate immune response are yet to be fully explored. This study aimed to assess the mode of action of trans-resveratrol in modulating the *P. gingivalis* LPS induced metabolic inflammation in the neuroblastoma IMR-32 in vitro cell model.

## Materials and methods

### Cell culture

The human neuroblastoma IMR-32 (ATCC) cell line was maintained in T25 flasks in Dulbecco’s modified Eagle’s medium (DMEM, Lonza UK) supplemented with 10% foetal bovine serum (FBS) Sigma-Aldrich, UK), 5 mM Pen/strep, 5 mM L glutamine, 5 mM sodium pyruvate (all from Gibco UK). The cell culture media was replaced every other day and incubated at 37 °C in a humidified atmosphere of 5% CO_2_, 95% air until they reached about 90% confluence. The confluent cells were detached using 1 mL of (1x) trypsin (Gibco UK)/flask and pelleted by centrifugation. The cell density was determined using a Countess® cell counting chamber slides (ThermoFisher, Scientific, UK).

IMR-32 cells are relatively slow-growing cells with a population doubling time of approximately 48 h. Fully confluent (> 90%) cells were used for PgLPS treatment in this study. For the treatment, the cells were seeded at 2 × 10^4^ cells/mL in a six-well plate. The cells reached a state of > 90% confluence within 6–7 days of seeding. On the day prior to treatment, the media was replaced with 1 mL serum and antibiotic-free media and cells were kept in the incubator for overnight. Then, cells were treated with 25 μM of 98% trans-resveratrol (Kingherbs Ltd. Hunan, China) from *P. cuspidatum* in the presence/absence of 10 µg/mL commercial PgLPS from *P. gingivalis* (tlrl-ppglps, InvivoGen, France). The concentration of trans-resveratrol (25 μM) was chosen based on the fact that resveratrol had no adverse effect on the cell viability and cytotoxicity up to 50 μM [19, 20]. The PgLPS dose was selected based on the fact that a concentration of 10 µg/ml PgLPS was reported to effectively induced intracellular oxidative and inflammatory cascades in murine macrophages [21]. The DMEM media without PgLPS served as a carrier control. Following 24 h incubation in the presence of PgLPS and resveratrol, the supernatants were removed and the cells were harvested in 1 ml Trizol solution and stored at − 20 °C until used for RNA extraction.

### RNA extraction

Total RNA was extracted from the cells using GenElute Total RNA Miniprep Kit (Sigma-Aldrich Corp.) according to the manufacturer's instructions. Total RNA was subjected to DNAse I (Sigma-Aldrich Corp.) treatment to eliminate genomic DNA contamination. Column purification of the RNA was performed using GenElute mammalian total RNA miniprep kit (Sigma-Aldrich Corp.). Total RNA was finally suspended in 50 μl 0.1% diethylpyrocarbonate (DEPC) treated water and stored at − 80 °C. The quality and quantity of the total RNA were assessed in a NanoDrop-ND1000 Spectrophotometer (Thermo Fisher Scientific Inc. MA, USA). The cDNA synthesis was performed with 1 μg of total RNA using the RevertAid H minus first-strand cDNA synthesis kit (Fermentas GmbH, St. Leon-Rot, Germany) following the manufacturer's protocol. The final volume of cDNA was adjusted to 150 μl with nuclease-free water.

### Quantitative real-time PCR (qPCR)

The expressions of a panel of 92 genes were evaluated using a qPCR array, performed in a QuantStudio 5 real-time PCR system (Applied Biosystems). This is a custom-designed 96-well array that included 92 target genes and four internal controls. The 92 target genes included representing the common markers of the inflammatory immune cascade. This panel of genes was previously reported to capture the intracellular signalling pathways associated with the chronic inflammation induced by LPS treatment [22]. Single internal control and 3 reference genes were also included on each plate. For this PCR array experiment, 25 μl cDNA (after 1:5 dilution) from each replicate within a single treatment group was pooled. QPCR was performed on a 20 μl reaction mixture per well containing 1 μl pooled cDNA, 9 μl water and 10 μl SYBR green master mix (Applied Biosystems). The thermal cycling conditions were 94 °C for 30 s followed by 60 °C for 1 min, for 40 cycles. In this experiment, a CT value of 35 was considered as the cut-off limit. The relative quantities (2^–ΔCt^) of the target genes were normalized using the geometric mean of the relative quantities of three internal control genes (beta-actin (*ACTB*), hypoxanthine–guanine phosphoribosyl transferase 1 (*HPRT1*) and beta-2 microglobulin (*B2M*). Briefly, average ΔCt was calculated as the difference of Ct values of any target gene minus the geometric average of the Ct value of the three reference genes. Then, fold change was calculated as 2^(− average ΔCt target gene)/^2^(− average ΔCt reference gene).^

### Pathway Analysis

The fold change values (cut off ± 1.5 fold) were analyzed using Qiagen Ingenuity Pathway Analysis and the relevant canonical pathways were identified using the default setting in the software. The statistical probability (*P* value) of the observed number of genes affecting a particular biological function was calculated based on Fisher's Exact Test. This *P* value indicated the statistical probability of the observed number of genes affected out of the total number of genes evaluated in the PCR array for the biological function. The correlation between the relationship direction and gene expression was determined by calculating the Z-score following the formula Z = (N_+_ − N_−_)/√ N where, N_+_ represents the number of genes whose expression follows the same direction while N_−_ represents the number of genes whose expression follows an opposite direction of the expression of a particular gene compared to that already available in the IPA knowledge database. N indicates the total number of genes affected. A high stringency Z-score between ≥  + 2.0 or ≤  − 2.0 were applied to identify the most relevant signalling pathways.

### Statistical analysis

This experiment involved technical replicates on three independent occasions and an equal volume of cDNA was pooled from each of the three replicates representing the treatment group, the relative mRNA abundance values for each pool was considered as the mean for the treatment group.

## Results

### Differential expression of genes by trans-resveratrol

**Fig. 1:**
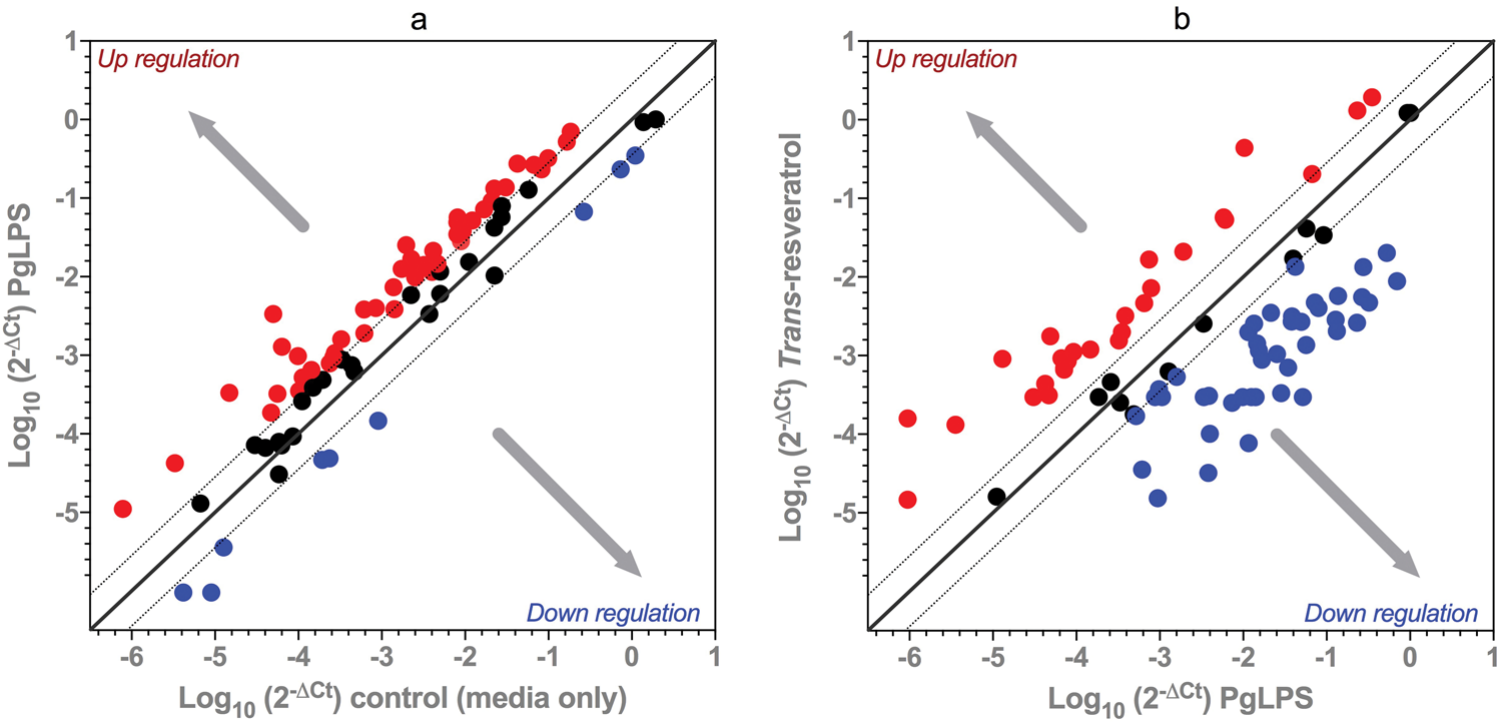
Relative abundance of mRNA transcripts of genes in the IMR-32 neuroblastoma cells subjected to *Porphyromonus gingivalis* lipopolysaccharide (PgLPS) treatment only (1a) or PgLPS and trans-resveratrol (1b)

Of the 92 target genes evaluated, compared to the carrier control the PgLPS treatment resulted in the relative abundance of mRNA transcripts increased for 49 genes, decreased for 7 genes and no alteration in 36 genes (Fig. 1a). Among the key genes up-regulated by > twofold (Table 1) were transcription factors (*FOXO1, STAT1, STAT3, CREB1, EGR2, IRF1, FOS, RELA, NFKB1*), kinases (*AKT1, PIK3R1, GSK3B, PCK1, CSF1R, IRAK1, JAK2, MAPK3K1, IKBKB, INSR*), receptors and associated protein (*VCAM1, MYD88, CD40, TNFRSF1A, IGF1R, PTGER1, HRH3*), enzymes (*PTGS2, IRS1, TRAF2, ADA, FTO, HADH*) and cytokines (*TNFA, IL1B, CSF1, CSF2, IL6, IL8* and *IL17A*). The 7 genes down-regulated by PgLPS were *GSTP1, SOD1, SOD2, TGFB1, RETN, ADIPOQ* and *LEPR*.

**Table 1:** Differential expression of genes caused by *Porphyromonus gingivalis* lipopolysaccharide (PgLPS) treatment only or PgLPS and trans-resveratrol in the IMR-32 neuroblastoma cells (fold change values ≥ 2.0 and ≤  − 2.0 folds are shown)

Genes	Location	Type of molecules	PgLPS	PgLPS + trans-resveratrol
MYD88 innate immune signal transduction adaptor (*MYD88*)	Plasma membrane	Adapter protein	+ 4.87	− 2.96
Tumor necrosis factor alpha (*TNFA*)	Extracellular space	Cytokine	+ 4.39	− 2.32
Interleukin 1 beta (*IL1B*)	Extracellular space	Cytokine	+ 4.61	− 3.06
Colony stimulating factor 1 (*CSF1*)	Extracellular space	Cytokine	+ 5.34	− 29.33
Interleukin 6 (*IL6*)	Extracellular space	Cytokine	+ 67.17	− 11.30
Interleukin 8 (*IL8*)	Extracellular space	Cytokine	+ 22.51	–
Colony stimulating factor 2 (*CSF2*)	Extracellular space	Cytokine	+ 3.10	+ 10.96
Interleukin 17A (*IL17A*)	Extracellular space	Cytokine	+ 2.60	+ 9.82
Prostaglandin-endoperoxide synthase 2 (*PTGS2*)	Cytoplasm	Enzyme	+ 2.49	− 2.71
Insulin receptor substrate 1 (*IRS1*)	Cytoplasm	Enzyme	+ 5.77	+ 4.78
Glutathione S-transferase pi 1 (*GSTP1*)	Cytoplasm	Enzyme	− 3.13	+ 5.51
TNF receptor associated factor 2 (*TRAF2*)	Cytoplasm	Enzyme	+ 4.16	− 12.25
Adenosine deaminase (*ADA*)	Cytoplasm	Enzyme	+ 4.71	− 39.38
FTO alpha-ketogluterate dependent dioxygenase (*FTO*)	Nucleus	Enzyme	+ 4.26	− 15.16
Superoxide dismutase 1 (*SOD1*)	Cytoplasm	Enzyme	− 3.98	+ 3.06
Superoxide dismutase 2 (*SOD2*)	Cytoplasm	Enzyme	− 3.21	+ 5.60
Hydroxyacetyle Co-A dehydrogenase (*HADH*)	Cytoplasm	Enzyme	+ 4.44	− 23.79
Prostaglandin E receptor 1 (*PTGER1*)	Plasma membrane	G-protein coupled receptor	+ 2.72	− 12.62
Histamine receptor H3 (*HRH3*)	Plasma membrane	G-protein coupled receptor	+ 6.25	− 119.21
Epidermal growth factor (*EGF*)	Extracellular space	Growth factor	–	+ 9.31
Insulin like growth factor 1 (*IGF1*)	Extracellular space	Growth factor	–	+ 8.78
Transforming growth factor beta 1 (*TGFB1*)	Extracellular space	Growth factor	− 4.84	+ 36.00
Vascular endothelial growth factor A (*VEGFA*)	Extracellular space	Growth factor	–	− 13.35
Phosphoenol pyruvate carboxylase 1 (*PCK1*)	Cytoplasm	Kinase	+ 2.38	+ 9.67
Colony stimulating factor 1 receptor (*CSF1R*)	Plasma membrane	Kinase	+ 3.44	+ 5.70
Interleukin 1 receptor associated kinase 1 (*IRAK1*)	Plasma membrane	Kinase	+ 3.26	− 68.45
Glycogen synthase kinase 3 beta (*GSK3B*)	Nucleus	Kinase	+ 4.40	− 2.70
Janus kinase 2 (*JAK2*)	Cytoplasm	Kinase	+ 3.59	− 61.71
Phoshpoinositide-3 kinase regulatory subunit 1 (*PIK3R1*)	Cytoplasm	Kinase	+ 6.08	− 5.28
Mitogen activated protein kinase kinase kinase 1 (*MAP3K1*)	Cytoplasm	Kinase	+ 5.16	− 6.10
Inhibitor of nuclear factor kappa B kinase subunit beta (*IKBKB*)	Cytoplasm	Kinase	+ 3.14	− 10.25
Interleukin 1 receptor associated kinase 2 (*IRAK2*)	Plasma membrane	Kinase	–	− 17.32
AKT serine/threonine kinase 1 (*AKT1*)	Cytoplasm	Kinase	+ 3.78	− 78.92
Insulin receptor (*INSR*)	Plasma membrane	Kinase	+ 4.38	− 47.27
Nuclear receptor subfamily 4 group A member 2(*NR4A2*)	Nucleus	Ligan-dependent nuclear receptor	+ 2.63	− 2.96
Dipeptidyl peptidase 4 (*DPP4*)	Plasma membrane	Peptidase	–	+ 12.00
Mucin 5AC, oligomeric mucus/gel-forming (*MUC5AC*)	Cytoplasm	Peptidase	–	+ 22.46
Plasminogen activator, tissue type (*PLAT*)	Extracellular space	Peptidase	+ 2.87	− 5.81
Insulin degrading enzyme (*IDE*)	Extracellular space	Peptidase	+ 7.03	− 41.87
Glucose-6 phosphatase catalytic subunit (*G6PC*)	Cytoplasm	Phosphatase	+ 20.16	− 2.03
Early growth response 2 (*EGR2*)	Nucleus	Transcription regulator	+ 3.31	+ 9.20
Interferon regulatory factor 1 (*IRF1*)	Nucleus	Transcription regulator	+ 2.32	− 151.71
Signal transducer and activator of transcription 1 (*STAT1*)	Nucleus	Transcription regulator	+ 4.08	− 14.09
cAMP responsive element binding protein 1(*CREB1*)	Nucleus	Transcription regulator	+ 6.13	− 18.41
Fos proto-oncogene, AP-1 transcription factor subunit (*FOS*)	Nucleus	Transcription regulator	+ 12.90	− 24.27
Forkhead box O1 (*FOXO1*)	Nucleus	Transcription regulator	+ 7.22	− 42.19
RELA proto-oncogene, NF-kB subunit (*RELA*)	Nucleus	Transcription regulator	+ 2.22	− 44.64
Nuclear factor kappa B subunit 1 (*NFKB1*)	Nucleus	Transcription regulator	+ 4.27	− 48.93
Signal transducer and activator of transcription 3 (*STAT3*)	Nucleus	Transcription regulator	+ 2.81	− 88.34
Sterol regulatory element binding transcription factor 1 (*SREBEF1*)	Nucleus	Transcription regulator	+ 4.23	− 174.32
Jun proto-oncogene, AP1 transcription factor subunit (*JUN*)	Nucleus	Transcription regulator	–	− 3.12
Interleukin 6 receptor (*IL6R*)	Plasma membrane	Trans-membrane receptor	+ 9.91	− 2.62
Leptin receptor (*LEPR*)	Plasma membrane	Trans-membrane receptor	− 2.17	+ 42.48
CD40 molecule (*CD40*)	Plasma membrane	Trans-membrane receptor	+ 4.53	+ 7.12
Vascular cell adhesion molecule 1 (*VCAM1*)	Plasma membrane	Trans-membrane receptor	+ 3.89	− 3.57
TNF receptor superfamily member 1A (*TNFRSF1A*)	Plasma membrane	Trans-membrane receptor	+ 2.88	− 19.77
Insulin like growth factor 1 receptor (*IGF1R*)	Plasma membrane	Trans-membrane receptor	+ 5.89	− 64.43
Uncoupling protein 2 (*UCP2*)	Cytoplasm	Transporter	+ 6.51	− 20.67
TNF receptor associated factor 5 (*TRAF5*)	Cytoplasm	Transporter	+ 3.91	− 33.05
Low density lipoprotein receptor (*LDLR*)	Plasma membrane	Transporter	+ 3.19	− 85.47
Nucleotide binding oligomerization domain containing 2 (*NOD2*)	Cytoplasm	–	–	+ 10.84
Resistin (*RETN*)	Extracellular space	–	− 6.15	+ 8.10
Adiponectin, C1Q and collagen domain containing (*ADIPOQ*)	Extracellular space	–	− 4.14	+ 6.66
GRB2 associated binding protein 1 (*GAB1*)	Cytoplasm	–	+ 7.40	− 18.91

Trans-resveratrol, in the presence of PgLPS challenge increased the mRNA transcript abundance of 19 genes, decreased of 44 genes with no alteration of 29 genes (Fig. 1b). Among the key 19 genes that were up-regulated by trans-resveratrol by a magnitude of > twofold (Table 1) include *ADIPOQ, IRS1, GSTP1, SOD1, SOD2, CSF1R, CD40, RETN, IGF1, EGR2, EGF, PCK1, IL17A, NOD2, CSF2, DPP4, MUC5AC, TGFB1* and *LEPR.* The 44 genes down-regulated by trans-resveratrol include transcription factors (*CREB1, FOXO1, FOS, IRF1, JUN, NFKB1, RELA, STAT1, STAT3* and *SREBEF1*), kinases (*AKT1, PIK3R1, GSK3B, IKBKB, INSR, IRAK1, IRAK2, JAK2* and *MAP3K1*), receptors (*IGF1R, MYD88, IL6R, HRH3, TNFRSF1A, PTGER1* and *VCAM1*), enzymes (*ADA, FTO, HADH, IDE, PLAT, PTGS2,* and *TRAF2*) and cytokines (*TNFA, IL6, IL1B* and *CSF1*).

### The biochemical pathways altered by trans-resveratrol in IMR-32 cells

Based on the expression of genes differentially expressed by the trans-resveratrol treatment in PgLPS challenged IMR-32 cells, the intracellular signalling pathways were identified (Table 2). Trans-resveratrol significantly (P < 0.001; z-score cut off < − 2.000) inhibited pathways of cellular oxidative stress including the production of NO and ROS as indicated by the lower mRNA abundance of *AKT1, FOS, IKBKB, IRF1, JAK2, MAPK3K1, NFKB1, PIK3R1, RELA, STAT1, TNFA* and *TNFRSF1* genes and iNOS signaling as indicated by a decrease in the mRNA abundance of *FOS, IKBKB, IRAK1, IRAK2, IRF1, JAK2, NFKB1, RELA* and *STAT1* genes. Trans-resveratrol also inhibited the major inflammatory signalling pathways such as NF-kB signalling (18 genes), neuro-inflammatory signalling (22 genes), acute phase response (18 genes), IL-6 pathways (15 genes), IL-8 pathways (12 genes) and CD40 pathways (10 genes). For the nutrient metabolic pathways, trans-resveratrol activated PTEN pathways (9 genes), PPARa/RXRa pathways (10 genes) and PPAR signalling pathways (10 genes) while inhibited IGF-1 pathways (9 genes) and insulin receptor pathways (8 genes).

**Table 2:** Canonical pathways and the gene affected by the trans-resveratrol in presence of *Porphyromonus gingivalis* lipopolysaccharide (PgLPS) treatment in the IMR-32 neuroblastoma cells

Pathways	Effect	Genes affected within the pathway	Z-score	P-value
*Oxidative stress*				
Production of NO and ROS	Inhibition	*AKT1, FOS, IKBKB, IRF1, JAK2, MAP3K1, NFKB1, PIK3R1, RELA, STAT1, TNF, TNFRSF1*	− 2.309	1.51E−13
iNOS signaling	Inhibition	*FOS, IKBKB, IRAK1, IRAK2, IRF1, JAK2, NFKB1, RELA, STAT1*	− 2.330	6.10E−17
*Inflammation*				
NF-kB signaling	Inhibition	*AKT1, CD40, EGF, GSK3B, IGF1R, IKBKB, IL1B, INSR, IRAK1, MAP3K1, MYD88, NFKB1, PIK3R1, RELA, TNFA, TNFRSF1A, TRAF2, TRAF5*	− 2.089	2.02E−27
Neuroinflammation signaling	Inhibition	*AKT1, CD40, CREB1, CSF1R, FOS, GSK3B, IDE, IKBKB, IL6, IL1B, IL6R, IRAK1, IRAK2, JAK2, MYD88, NFKB1, PIK3R1, PTGS2, RELA, SOD2, STAT1, TGFB1*	− 2.000	2.70E−32
Acute phase response	Inhibition	*AKT1, FOS, IKBKB, IL6, IL1B, IL6R, IRAK1, JAK2, MAP3K1, MYD88, NFKB1, PIK3R1, RELA, SOD2, STAT3, TNF, TNFRSF1A, TRAF2*	− 2.186	9.55E−24
IL-6 signaling	Inhibition	*AKT1, FOS, IKBKB, IL6, IL1B, IL6R, JAK2, NFKB1, PIK3R1, RELA, STAT3, TNF, TNFRSF1A, TRAF2, VEGFA*	− 2.324	3.89E−21
IL-8 signaling	Inhibition	*AKT1, EGF, FOS, IKBKB, IRAK1, IRAK2, NFKB1, PIK3R1, PTGS2, RELA, VCAM1, VEGFA*	− 2.111	2.75E−13
CD40 signaling	Inhibition	*CD40, FOS, IKBKB, NFKB1, PIK3R1, PTGS2, RELA, STAT3, TRAF2, TRAF5*	− 2.333	1.61E−15
*Metabolism*				
PTEN signaling	Activation	*AKT1, FOXO1, GSK3B, IGF1R, IKBKB, INSR, NFKB1, PIK3R1, RELA*	2.121	6.43E−11
PPARa/RXRa activation	Activation	*ADIPOQ, IKBKB, IL6, IL1B, INSR, IRS1, JAK2, NFKB1, RELA, TGFB1*	2.111	4.61E−12
PPAR signaling	Activation	*FOS, IKBKB, IL1B, INSR, NFKB1, PTGS2, RELA, TNF, TNFRSF1A, TRAF2*	2.001	4.26E−15
IGF-1 signaling	Inhibition	*AKT1, FOS, FOXO1, IGF1, IGF1R, IRS1, JAK2, PIK3R1, STAT3*	− 2.121	2.63E−13
Insulin receptor signaling	Inhibition	*AKT1, FOXO1, GAB1, GSK3B, INSR, IRS1, JAK2, PIK3R1*	− 2.017	1.97E−10

## Discussion

Resveratrol is a bioactive polyphenol that has numerous health benefits including immuno-modulatory properties. The LPS of *P. gingivalis* (PgLPS), an anaerobic pathogen, implicated in periodontal disease induces innate immune responses that have far-reaching consequences in the immune and metabolic health of the host. In this study, the transcript abundance of a panel of key biomarkers altered by trans-resveratrol in the presence of PgLPS in IMR-32 neuroblastoma cells was evaluated. Gene expression analysis revealed that the markers of cellular oxidative stress, inflammatory pathways and glucose homeostasis as induced by the PgLPS treatment were altered by trans-resveratrol. This study demonstrates the potential of trans-resveratrol as a bioactive compound that has multiple modes of action including therapeutic potential in some neuroinflammatory diseases.

*P. gingivalis* and its LPS are predominantly found in the oral cavity, potentially contributing to dementing diseases of late-onset [1, 2, 7]. In Alzheimer’s patients, the circulating plasma level of LPS was reported to be 61 ± 42 pg/ml versus 21 ± 6 pg/ml in the healthy volunteers [23]. In cell culture experiment, even a concentration of 10 ng/ml PgLPS caused only a mild response, however, a higher concentration of PgLPS (10 µg/ml) effectively induced intracellular oxidative and inflammatory cascades in murine macrophages [21]. Hence, PgLPS 10 µg/ml was used to treat the cells in this experiment. In this study, PgLPS treatment of IMR-32 cells resulted in a conspicuous up-regulation of key signalling molecules including transcription factors, kinases, membrane receptors, growth factors, enzymes and cytokines. Taken together, this data indicates the plausible activation of the oxidative and inflammatory cascades.

An LPS mediated activation of pro-inflammatory response is expected to occur through the involvement of pathogen recognition receptors (PRRs) molecules such as the toll-like receptors 4 (TLR-4) and 2 (TLR-2) and via MyD88 route leading to the activation of NF-kB [24]. This was evident from the increase mRNA abundance of MyD88, a number of genes of NF-kB pathway as well as major downstream inflammatory cytokines including TNFa, IL6 and IL1B indicating that PgLPS indeed induced the inflammatory signal cascade in IMR-32 cells. In periodontal inflammation, PgLPS was reportedly increased the levels of major inflammatory cytokines including TNFa, IL-6, IL-1B and IL-17 [25, 26]. These pro-inflammatory cytokines can potentially contribute to the development of a systemic chronic inflammatory condition and pathophysiology of other inflammatory diseases such as cardiovascular diseases and type-2 diabetes [3, 5]. Thus, an unhygienic oral environment with generalised periodontitis may negatively affect a number of human inflammatory, but chronic diseases because of the common signalling cascades being activated. The drive to combat chronic diseases, which lower the individual’s quality of life, is to improve lifestyle habits [27, 28].

In this study, the anti-oxidative and anti-inflammatory effects of trans-resveratrol were evident from the down-regulation of a number of key regulatory kinases, trans-membrane receptors, enzymes and inflammatory cytokines all together indicating a potential therapeutic application of resveratrol in *P. gingivalis* mediated pro-inflammatory disease that may eventually lead to AD. Resveratrol (3,5,4′-trihydroxy-trans-stilbene), a polyphenol naturally present in many plant components and long known for its anti-oxidative, anti-inflammatory properties and its role in glucose metabolism, has been used in the oriental medicine for centuries [29]. We identified that the anti-oxidative activity of trans-resveratrol is mediated through inhibition of two vital oxidative stress pathways (production of nitric oxide (NOS) and reactive oxygen species (ROS) and iNOS signalling pathways. ROS are oxygen-containing molecules produced as a result of cellular metabolism and are required for facilitating the transfer of electrons in the redox reactions as well as serving as a second messenger [30]. Uncontrolled oxidative stress is associated with metabolic inflammation as well as neurodegenerative diseases such as AD [31, 32]. In AD, ROS interacting with Aβ leads to the production and accumulation of toxic compounds and plaque formation ultimately leading to synaptic loss and a compromised cognitive function in AD pathogenesis [30, 32]. Another mechanism of ROS in AD pathogenesis involves its contribution towards the progressive loss of neuronal autophagy, a vital mechanism through which cell degrades the cytoplasmic proteins and organelles [30, 33].

Underlying bioactivity of trans-resveratrol could be mediated via the inhibition of phosphor-inositol 3 kinase (PI3K)/AKT1 pathway because the mRNA abundance of PI3KR1, a regulatory PI3K, along with AKT1, a serine/threonine kinase were inhibited by trans-resveratrol in the presence of PgLPS. The PI3K/AKT1 pathway plays an important role in the cellular oxidation process and contributes to the pathophysiology of neurodegenerative diseases [34]. While the PI3K/AKT pathway is indispensable for maintaining the homeostatic level of cell growth, survival and differentiation and metabolism of nutrients [34], during the cellular oxidative stress PI3K together with AKT1, trigger the production of ROS [35, 36]. AKT1 is involved in the phosphorylation of a number of signalling molecules such as glycogen synthase kinases 3 beta (GSK-3B), forkhead box class O1 (FOXO1) and NF-kB [34]. Along with PI3KR1 and AKT1, the mRNA abundance of these markers was also down-regulated by trans-resveratrol in this experiment. An LPS-mediated inflammatory response mediated through its receptor molecule TLR4 is also associated with the activation of AKT1 [37]. However, in this experiment, no change in the TLR4 mRNA was evident while cells were treated with trans-resveratrol in the presence of PgLPS. A potentially underlying cause could be that because the cells were harvested 24 h post-treatment, a time scale regarded sufficient to dampen the TLR4 expression caused by a higher level of AKT1 expression over time [38].

The bioactivities of trans-resveratrol are known to affect the oxidative stress pathways, systemic inflammatory signalling and glucose metabolic pathways [39, 40]. Such broad-spectrum bioactivities of trans-resveratrol was exhibited by affecting the GSK-3B, an important regulatory molecule involved in the phosphorylation of a number of proteins and transcription factors involved in these three interconnected pathways [34, 40]. In addition to the PgLPS mediated inflammatory process, GSK-3B also contributes to excessive phosphorylation of the microtubule binding protein tau which eventually caused their collapse leading to neurofibrillary tangle formation, which is the second diagnostic hallmark of AD neuropathology [30, 41]. Another mechanism through which GSK-3B promotes the inflammatory process is through phosphorylation of STAT3, an important transcription factor required for the induction of pro-inflammatory cytokines that translocate into the nucleus following phosphorylation and activation [41]. Transcription factor CREB1 involved in the TLR mediated inflammation serves as a substrate for GSK-3B [40]. The importance of GSK-3B molecule in LPS mediated inflammatory response was demonstrated by the fact that inhibition of GSK-3B effectively saved mice from the deleterious effect of a lethal doze of LPS [42]. In this experiment together with GSK3B, the mRNA abundance of STAT3 and CREB1 were also down-regulated indicating the therapeutic application of trans-resveratrol in PgLPS mediated deleterious pro-inflammatory response.

GSK-3B also plays a major role in the nutrient metabolism through directly interacting with insulin and mTOR molecules and directing the nutrient energy towards the anabolic path leading to cell growth [40]. Expression of GSK-3B is vital for homeostasis as the complete elimination of GSK-3B is fatal for cell survival as demonstrated in the knockdown embryonic model [42]. In contrast, a temporary inhibition was found to increase in glucose uptake as well as a higher expression of GLUT1 [43]. GSK-3B also functions as a negative regulator of mitochondrial energy production that is mediated through controlling the AMPK, a cellular energy-sensing molecule, and inhibition of pyruvate dehydrogenase activity [44]. In this experiment, while the mRNA levels of G6P, an entry point of glucose into the energy metabolism and mitochondrial uncoupling protein 2 (UCP2) were inhibited by trans-resveratrol; two other markers of glucose metabolism (IRS1 and PCK1) remained unaffected. This potentially complex mechanism of trans-resveratrol action in nutrient metabolism via affecting GSK-3B warrants further investigation.

FOXO1 belongs to the FoxO, forkhead box class O (FoxO) cluster of transcription factors that phosphorylates and acetylates proteins at serine, threonine and lysine residues and is involved in the oxidative stress and insulin action [34, 45]. Both insulin resistance and oxidative stress can enhance the transcriptional activity of FOXO1 that can again contribute to the development of hyperglycemia and the production of ROS [46]. FOXO1 can also activate c-Jun N-terminal kinase and inhibit Wingless (Wnt) pathways and can contribute to β-amyloid plaque formation and phosphorylation of s protein potentially leading to neurodegeneration [46]. In response to the *P gingivalis* infection that often results in a compromised barrier function of the mucosal/gingival epithelium, the PgLPS is expected to increase the FOXO1 expression [45]. Then, a subsequent translocation of FOXO1 into the nucleus is expected to cause transcriptional activation of a number of inflammatory mediators bearing a FOXO1 promoter responsive element [45]. FOXO1 activates a number of downstream molecules involved in the wound healing process of the epithelial barrier [47]. In this experiment, in addition to a reduction in the mRNA abundance of FOXO1, trans-resveratrol also down-regulated the abundance of few interesting downstream molecules such as pro-inflammatory cytokines (IL1B, TNFA), wound healing factor (VEGF) and adhesion molecule (VCAM1). TGFB is another FOXO1 target molecule that plays a vital role in the wound healing process [45]. Trans-resveratrol increased the mRNA abundance of TGFB suggesting its beneficial role in the PgLPS induced inflammatory damage.

Another interesting molecule with multiple protective functions in cell survival and metabolism is the phosphatase and tensin homolog (PTEN), a tumor suppressor that antagonizes phosphatidylinositol 3-kinase type I (PI3K) and thus affecting PI3K/AKT pathways [48]. PTEN is a tumour suppressor molecule that catalyses converting of PIP3 to PIP2 and reverses the PI3K/AKT activities. PTEN can increase energy expenditure in brown adipose tissue and enhance longevity at organism level indicating that an increase in PTEN activity could be beneficial [49]. One of the bioactivity of trans-resveratrol includes up-regulation of the PTEN pathway [48] that is associated with concomitant inhibition of PI3K/AKT functions. An outcome of this response is an up-regulation of genes involved in triggering the antioxidant pathways such as catalases and superoxide dismutase enzymes [48]. Our pathway analysis identified the activation of PTEN pathways as a potential mechanism of underlying anti-oxidant bioactivities of trans-resveratrol which is also conspicuous in the down-regulation of the mRNA abundance of major genes of PTEN pathway including AKT1, FOXO1, GSK3B, PIK3R1 and NF-kB associated markers. Besides, a potential triggering of genes involved in the anti-oxidant mechanism such as SOD1, SOD2 and GSTP1 [50] was also evident in this study further demonstrating multicomponent mechanisms underlying the trans-resveratrol mediated bioactivity in IMR-32 cells.

In conclusion, this study of targeted transcriptome profiling identified a number of biochemical signalling pathways affected in the IMR-32 neuroblastoma cells when treated with PgLPS. The gene expression analysis revealed that the trans-resveratrol has multiple highly effective bioactivities that work through modulating the interconnected mechanisms of cellular oxidative stress, inflammatory response and nutrient metabolism. Hence, the therapeutic application of trans-resveratrol in *P. gingivalis* mediated disease pathophysiology may be explored. Based on the altered expression of multiple markers of cellular oxidative stress, inflammatory pathways and nutrient metabolism, the PgLPS treated IMR-32 cells may be a good in-vitro model of *P. gingivalis* induced innate immune cascade that could eventually lead to the development of AD.
